# An exploratory data analysis of electroencephalograms using the functional boxplots approach

**DOI:** 10.3389/fnins.2015.00282

**Published:** 2015-08-19

**Authors:** Duy Ngo, Ying Sun, Marc G. Genton, Jennifer Wu, Ramesh Srinivasan, Steven C. Cramer, Hernando Ombao

**Affiliations:** ^1^Department of Statistics, University of California, IrvineIrvine, CA, USA; ^2^Computer, Electrical and Mathematical Sciences & Engineering Division, King Abdullah University of Science and TechnologyThuwal, Saudi Arabia; ^3^Department of Anatomy & Neurobiology, University of California, IrvineIrvine, CA, USA; ^4^Department of Cognitive Sciences, University of California, IrvineIrvine, CA, USA

**Keywords:** EEGs time series, functional boxplots, surface boxplots, spectral analysis, band depth, exploratory analysis, stationarity

## Abstract

Many model-based methods have been developed over the last several decades for analysis of electroencephalograms (EEGs) in order to understand electrical neural data. In this work, we propose to use the functional boxplot (FBP) to analyze log periodograms of EEG time series data in the spectral domain. The functional bloxplot approach produces a median curve—which is not equivalent to connecting medians obtained from frequency-specific boxplots. In addition, this approach identifies a functional median, summarizes variability, and detects potential outliers. By extending FBPs analysis from one-dimensional curves to surfaces, surface boxplots are also used to explore the variation of the spectral power for the alpha (8–12 Hz) and beta (16–32 Hz) frequency bands across the brain cortical surface. By using rank-based nonparametric tests, we also investigate the stationarity of EEG traces across an exam acquired during resting-state by comparing the spectrum during the early vs. late phases of a single resting-state EEG exam.

## 1. Introduction

Electroencephalograms (EEGs) have been used for many decades to study the complex spatio-temporal dynamics of brain processes (Nunez and Srinivasan, 2006). Due to its excellent temporal resolution (sampling rates usually range from 100 to 1000 Hz), EEGs can capture transient changes in brain activity, identify oscillatory behavior and study cross-dependence between EEG components. Since EEGs indirectly measure neuronal electrical activity, they can be used to infer the statistical properties of the underlying brain stochastic process. One such statistical property is the spectrum (or power spectrum) which decomposes the total variability in the EEG according to the contribution of oscillations at different frequencies. Most approaches to analyzing EEGs focus immediately on statistical modeling and spectral estimation. Here, we offer a systematic framework for exploring structures, patterns and features in the signal—prior to formal modeling. We explore the spectral properties only in a single channel using EEG traces from several epochs.

One approach to estimating the spectrum using EEG traces is to fit a parametric time domain model, such as the autoregressive moving average (ARMA) model. Applications of parametric modeling of EEGs have a long history. See (Bohlin, 1973; Isaksson et al., 1981; Krystal et al., 1999; Jain and Deshpande, 2004) among many others. When the spectrum of the EEG evolves over time (e.g., within an epoch), one could still use the ARMA model but allow the coefficients to vary over time. A key element in ARMA models is the order of the autoregressive (AR) and moving average (MA) components. These can be obtained objectively using an information-theoretic criterion such as the Akaike information criterion (AIC) and the Bayesian information criterion (BIC). Using these criteria, we obtain an optimal AR and MA order that jointly gives the best fit with the least complexity (as determined by the orders). BIC puts a heavier penalty for complexity compared to AIC and thus often gives a model with lower orders (lower complexity). From the parametric fit, we derive the estimates of the auto-correlation function and the spectrum. The theoretical background for parametric models are developed in Priestley (1981), Shumway and Stoffer (2000), and Brockwell and Davis (2009).

One could also estimate the spectrum without resorting to a parametric model. Under this approach, the EEGs are considered to be superpositions of sines and cosines (Fourier waveforms) with different frequencies and random amplitudes. These random amplitudes (or coefficients) are computed using the fast Fourier transform (FFT). The squared magnitude of these amplitudes, often called the periodograms, are the data-analogs of the spectrum defined on discrete frequencies. The theoretical background on the frequency domain approach to time series is developed in Brillinger (1981) and Percival and Walden (1993). This approach to analyzing EEGs continues to be popular in the cognitive and brain sciences. The following papers cover both methods and applications of spectral analysis to EEGs: Pfurtscheller and Aranibar (1979), Bressler and Freeman (1980), Makeig (1993), and Srinivasan and Deng (2012), to name a few.

The common practice prior to spectral estimation is to pre-process EEGs, often to remove artifacts (Makeig et al., 1996). After artifact rejection and segmentation according to epochs, the spectrum is estimated from each EEG trace. As noted, there is a lack a systematic framework for exploring structures, patterns and features in the signal—prior to formal modeling. Due to the complexity of EEG data, exploratory data analysis (EDA) plays an important role, especially when data are recorded from many epochs or trials during an experiment. For example, it is often expected that brain responses to the same stimulus ought to be relatively uniform, with minimal variation across epochs. In contrast, greater variability across epochs may be expected during neuroimaging studies that examine the brain in resting-state, as cognitive processes can vary within and across sessions for individual subjects and across subjects. An appropriate EDA methods can provide insights into features of EEG, including similarities and variability of the brain responses across epochs to facilitate the statistical model. In this paper, we propose to use the functional boxplot (FBP) method originally developed by Sun and Genton (2011) to address these questions.

**Figure 1:**
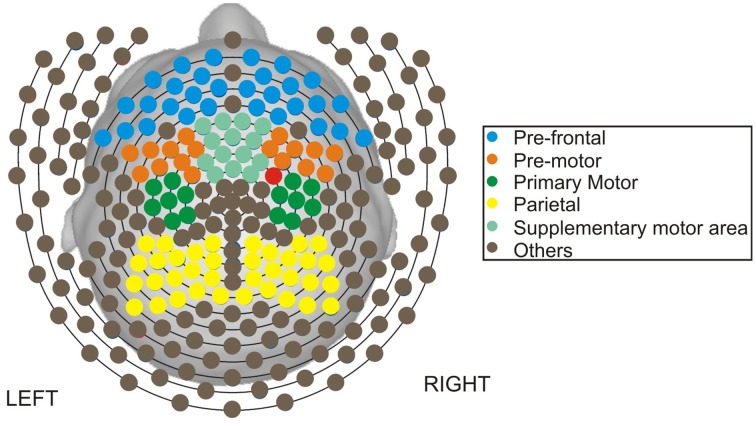
**Map of channels on the scalp**.

The methods presented in this paper are motivated by a motor skill acquisition study at the Neuro-rehabilitation laboratory at the University of California, Irvine (Principal Investigator: Steven C. Cramer). In the previous study, EEG was recorded from 17 subjects both during resting-state prior to motor skill training and during motor skill training using dense-array EEG (256 electrodes) as shown in Figure 1. The resting-state EEG exam was 3 min, and during post-processing, was segmented into 1-s non-overlapping epochs. As demonstrated in Wu et al. (2014), the spectral features of the resting-state EEGs when combined with a partial least squares regression analysis, was predictive of an individual's subsequent ability to acquire a novel motor skill. These may be of clinical importance to the field of rehabilitation, as improved methods for stratifying patients may significantly improve response to treatment and assist allotment of limited resources.

We present an *exploratory spectral analysis* (ESA) of resting-state EEG traces using FBPs for one subject. In spectral analysis, the spectrum is an important stochastic property of the signal. It indicates the amount (or proportion) of variance that is explained by each frequency bin. Thus, the spectrum or the log spectrum of the EEG signal can be used to examine relative amounts of variability explained by slow (delta or theta) waves and fast (alpha or beta) waves. Throughout this analysis, we obtain a *sample* spectral curve by smoothing the log periodograms of each 1-s EEG epoch, and treat it as one observation unit in the FBP. By using the FBP, we address three primary objectives. The *first* objective is to identify the median, i.e., the most characteristic spectral curve rather than the pointwise frequency-specific medians. In addition, outliers are demonstrated by their unusual sample log spectral curve, and can be caused by extra-brain artifacts, including eye blinks, eye movements, and muscle movements in the EEG signal. Subsequently, confirmed outliers will be removed from subsequent analyses. The advantage of the FBP approach, over the usual pointwise boxplot method, is that it identifies epochs that have potential outlying spectral curves.

The *second* objective is to compare the median curves and the variability of the spectral curves from multiple phases of the resting state period. To test the stationarity of the EEG signal over the entire recording, we compare the spectral curves and the frequency-specific spatial distribution of spectral power during the early phase (first 60 epochs) vs. the late phase (last 60 epochs). Evidence against stationarity must be taken seriously since this would suggest an evolution of brain processes across the recording (Fiecas and Ombao, under revision). Moreover, the FBP approach is able to provide some characterization of the variation of the sample log spectral curves across EEG recording. In experiments comparing more than one group (e.g., healthy controls vs. patients with stroke), it would be also interesting to determine whether groups differ with respect to consistency (uniformity) of the EEG signal over time.

The *third* objective is to investigate the spatial variability of spectral power across the brain for a given frequency band using the surface boxplot, which is a generalization of the FBP. Using the surface boxplots approach, it is possible to identify cortical regions (or channels) that, relative to the other channels, exhibit a high proportion of beta power. The beta band is particular interest to neuroscientists, as changes in beta activity have a good association with motor function (Roopun et al., 2006; Joundi et al., 2012).

The remainder of the paper is organized as follows. In Section 2, we present a comprehensive exploratory method which consists of the following: a review of the spectra in Section 2.1, a demonstration of automatic bandwidth selector for periodogram smoothing using the gamma generalized crossvalidation criterion in Section 2.2, some remarks on smoothing the periodogram in Section 2.3, a description of the FBPs in Section 2.4, a description of the surface boxplots in Section 2.5, and a demonstration of testing for differences in mean curves between families of curves in Section 2.6. In Section 3, we examine the finite sample performance of the proposed exploratory method. In Section 4, the resting-state EEG data are analyzed. Finally, in Section 5, conclusions and future work are discussed.

## 2. Method for exploratory spectral analysis (ESA)

In this section, we review the methods that are needed for ESA of the EEG data. In Section 2.1, we first formally define the spectrum and then discuss a consistent estimator which is obtained by smoothing the periodogram using a bandwidth that is automatically selected by the gamma generalized cross-validation (Gamma-GCV) method described in Section 2.2. Next, we highlight two remarks on smoothing the periodogram in Section 2.3, then we present the FBPs method in Section 2.4 and surface boxplots method in Section 2.5. Finally, we present a rank sum test which tests for differences in median curves or surfaces between families of curves or surfaces in Section 2.6.

### 2.1. Spectrum

The spectrum of an EEG signal (which is assumed to be stationary) can give the amount of variance contributed by oscillatory components (from delta to beta band activity). Let *X*(*t*), *t* = …, −1, 0, 1, … be a zero-mean stationary time series with covariance function γ(τ) = *E*(*X*(*t*)*X*(*t* + τ))(τ = …−1, 0, 1, …) that is assumed to be absolutely summable, i.e., $\overset{\infty }{\underset{\tau =-\infty }{\sum }}|\gamma \left(\tau \right)|<\infty$. The spectrum, denoted *f*(ω), is defined to be

$$f(\omega )=\sum_{\tau =-\infty }^{\infty }\gamma (\tau )\;e^{-i2\pi \omega \tau },\;\omega \in \left[-\frac{1}{2},\frac{1}{2}\right].$$

The starting point for estimating *f*(ω) is the periodogram. Denote *I*(ω_*k*_) to be the periodogram computed from a finite sample of the stationary process *X*(0), *X*(2), …, *X*(*T* − 1) at frequency ω_*k*_ = *k*∕*T* which is defined to be

$$I(\omega_{k})=\frac{1}{T}{\left|\sum_{t\;=\;0}^{T-1}X(t)\;e^{-i2\pi \omega_{k}t}\right|}^{2},\;k=-[\text{​}[T/2]\text{​}]-1,\ldots ,[\text{​}[T/2]\text{​}],$$

where 〚T/2〛 is the quotient of *T*∕2.

**Figure 2:**
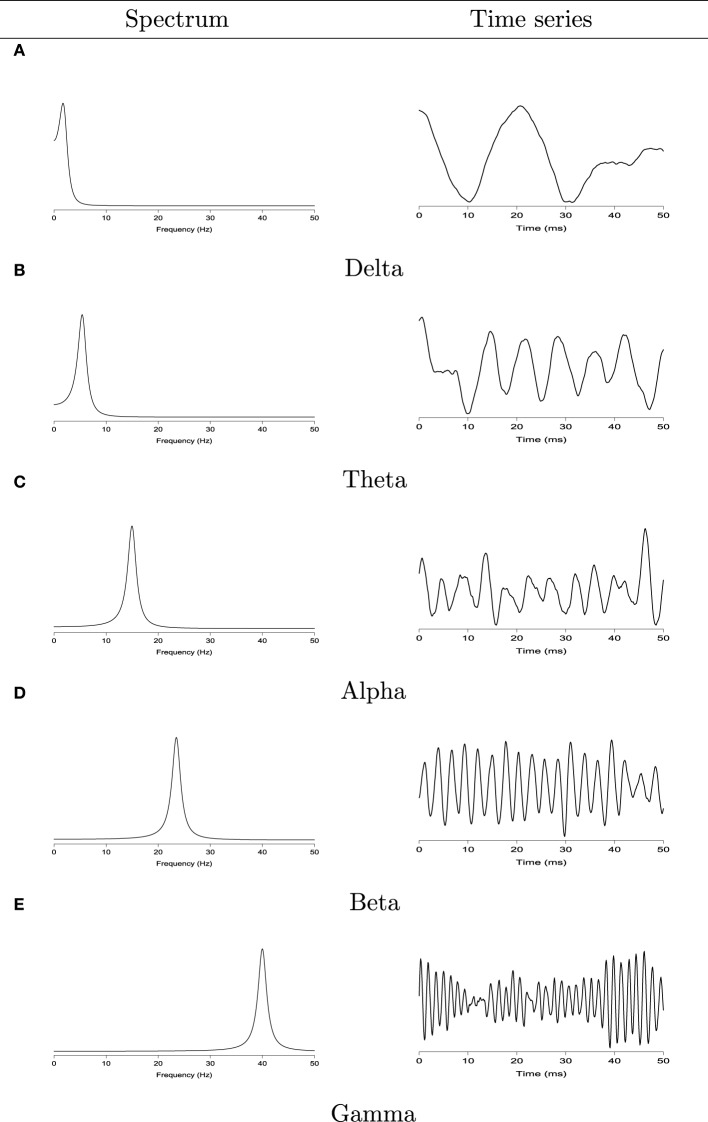
**Left:** the spectrum of second order auto-regressive processes AR(2) with power concentrated at the delta (0–4 Hz), theta (4–8 Hz), alpha (8–16 Hz), beta (16–32 Hz), and gamma (32–50 Hz) bands. **Right**: realizations from each corresponding AR(2) process. **(A)** Delta band. Left: the spectrum of second order auto-regressive processes AR(2). Right: realizations from each corresponding AR(2) process. **(B)** Theta band. Left: the spectrum of second order auto-regressive processes AR(2). Right: realizations from each corresponding AR(2) process. **(C)** Alpha band. Left: the spectrum of second order auto-regressive processes AR(2). Right: realizations from each corresponding AR(2) process. **(D)** Beta band. Left: the spectrum of second order auto-regressive processes AR(2). Right: realizations from each corresponding AR(2) process. **(E)** Gamma band. Left: the spectrum of second order auto-regressive processes AR(2). Right: realizations from each corresponding AR(2) process.

To characterize the spectra of the EEG signals, we classify the oscillatory patterns of periodograms into four primary frequency bands: delta (0–4 Hz), theta (4–8 Hz), alpha (8–16 Hz), beta (16–32 Hz), and gamma (32–50 Hz) as shown in Figure 2. Since each frequency band is defined by a range, we define $\hat{S}\left(\Omega \right)$ to be the estimated spectral power at the Ω band:

$$\hat{S}(\Omega )=\int_{\omega \in \Omega }I(\omega )d\omega .$$

It is well-known that the periodogram *I*(ω_*k*_) is an asymptotically unbiased estimator for *f*(ω_*k*_), but it is inconsistent because its variance approaches a positive constant when *T* → ∞. Therefore, to reduce the variance, we smoothed the periodogram. A number of nonparametric smoothing methods have been proposed including the kernel smoother (Lee, 1997; Ombao et al., 2001), wavelet (Gao, 1997), smoothing spline (Wahba, 1980; Pawitan and O'sullivan, 1994), or local polynomial (Fan and Kreutzberger, 1998). For kernel smoothing, Ombao et al. (2001) developed an automatic span selector via the generalized crossvalidation criterion for generalized additive models based on the *deviance* which is discussed in Section 2.2.

### 2.2. Automatic span selector using the gamma generalized crossvalidation method

From Brillinger (1981) (Theorem 5.2.6), *I*(ω_*k*_) follows an asymptotic distribution

$$I(\omega_{k})\sim \{\begin{array}{ll} Gamma(1,f(\omega_{k})) & k=1,\ldots ,T/2-1\\ Gamma(\frac{1}{2},2f(\omega_{k})) & k=0,T/2, \end{array}$$

where *I*(ω_0_), …, *I*(ω_*T*∕2_) are independent. As a caveat, we note here that the actual result requires that the number of frequencies is fixed and does not depend on *T*. However, in most applications, this is often ignored. This result can be equivalently stated as *I*(ω_*k*_)∕*f*(ω_*k*_) ~ ϵ_*k*_ where $\epsilon_{k}\;\overset{{}^{\circ}}{\sim }\;\chi^{2}\left(1\right)$ when *k* = 0 or *T*∕2 and $\epsilon_{k}\;\overset{{}^{\circ}}{\sim }\;\frac{1}{2}\chi^{2}\left(2\right)$ when *k* = 1, …, *T*∕2 − 1. As noted, we need to smooth the periodogram *I*(ω_*k*_) to produce a consistent estimator for *f*(ω_*k*_). Let $\hat{f_{p}}\left(\omega_{k}\right)$ be a smoothed periodogram estimator of *f*(ω_*k*_) which we define to be

$$\hat{f_{p}}(\omega_{k})=\sum_{j=-p}^{p}W_{p,j}I(\omega_{k+j})\;k=0,\ldots ,T/2,\text{and }j=-p,\ldots ,p$$

where 2*p* + 1 is the smoothing span and *W*_*p, j*_ are nonnegative weights that satisfy the following conditions for any fixed *p*:

$$W_{p,j}=W_{p,-j}(j=1,\ldots ,p),\sum_{j=-p}^{p}W_{p,j}=1.$$

Generally, the weights are chosen so that *W*_*p, j*_ is a decreasing function of *p*, but (Priestley, 1981) shows that the choice of the weights *W*_*p, j*_ is of secondary importance to the value of the span or bandwidth. Thus, for simplicity, we use the boxcar smoother with weights defined by *W*_*p, j*_ = 1∕(2*p* + 1) for all *j* = −*p*, …, *p*. The gamma generalized crossvalidation method selects *p* to minimize the generalized crossvalidated deviance function

$$GCV(p)=\frac{M^{-1}\sum_{j=0}^{M-1}D(I(\omega_{j}),{\hat{f}}_{p}(\omega_{j}))}{{\left(1-\text{tr}(H_{p})/M\right)}^{2}},$$

where *M* = *T*∕2+1. The deviance $D\left(I\left(\omega_{j}\right),{\hat{f}}_{p}\left(\omega_{j}\right)\right)$ can be chosen as $q_{j}\left\{-\log\left(I\left(\omega_{j}\right)∕{\hat{f}}_{p}\left(\omega_{j}\right)\right)+\left(I\left(\omega_{j}\right)-{\hat{f}}_{p}\left(\omega_{j}\right)\right)∕{\hat{f}}_{p}\left(\omega_{j}\right)\right\}$ (McCullagh and Nelder, 1989). Here, *q*_*j*_ = 1 − 0.5ℐ{*j* = 0, *M* − 1}, and ℐ is the indicator function. The *H*_*p*_ is the smoother matrix with smoothing parameter *p*, and the term ${\left(1-tr\left(H_{p}\right)∕M\right)}^{2}$ often referred to as the model degrees of freedom, can be expressed in terms of the weight at the center of the smoothing window: ${\left(1-W_{p,0}\right)}^{2}$. Then, the generalized crossvalidated deviance function can be written as

$$\begin{array}{l} GCV(p)=\\ M^{-1}\sum_{j=0}^{M-1}q_{j}\left\{\frac{-\log(I(\omega_{j})/{\hat{f}}_{p}(\omega_{j}))+(I(\omega_{j})-{\hat{f}}_{p}(\omega_{j}))/{\hat{f}}_{p}(\omega_{j})}{{\left(1-W_{p,0}\right)}^{2}}\right\}. \end{array}$$

### 2.3. Remarks

**Figure 3:**
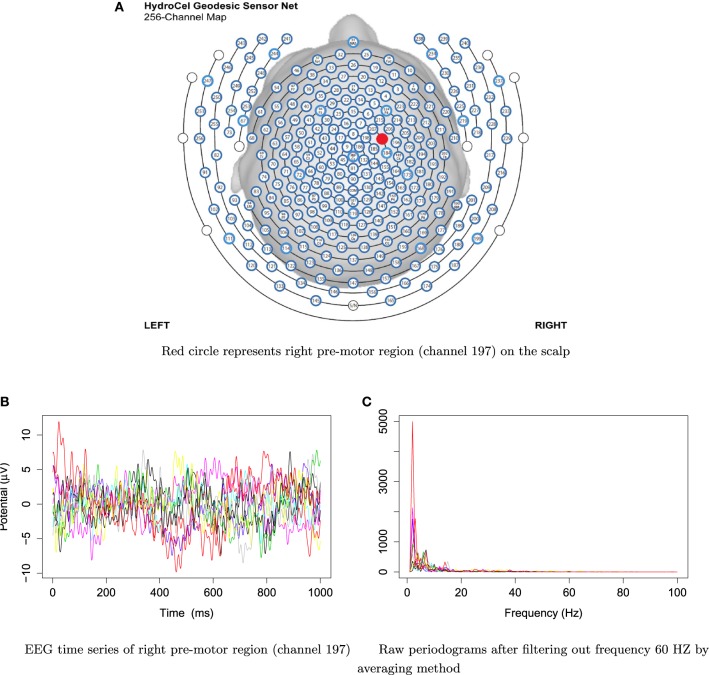
**EEG time series and raw periodograms after filtering out frequency 60 HZ by averaging method of channel 197 (right pre-motor region) for the first 10 traces**.

**Figure 4:**
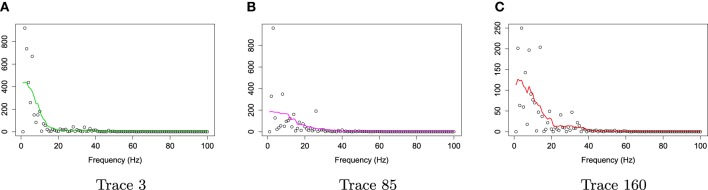
**Smoothing periodograms at randomly selected epochs 3, 85, and 160 of channel 197 (in the right pre-motor region) using the bandwidth that was automatically selected by the gamma generalized crossvalidation (gamma-GCV) method**. **(A)** Smoothing periodograms at epoch 3. **(B)** Smoothing periodograms at epoch 85. **(C)** Smoothing periodograms at epoch 160.

For frequencies over 100 Hz, the periodogram values are almost negligible because the signals underwent low–pass filtering at 100 Hz., so for simplicity, we will only show the spectrum over the frequency range of 0–100 Hz. In Figure 3, we show the location of channel 197 in right pre-motor region at the resting-state. Figure 4 gives an illustration of smoothing the periodograms for randomly selected epochs 3, 85, and 160 for a fixed channel 197. It can be seen that the power at these periodograms are dominated by low frequencies, and the values of smoothing span minimizing the generalized crossvalidated deviance function are about 3–5. Also, the smoothing lines reasonably approximate the periodograms and the small bandwidths preserve the peaks. Second, since the distribution of *I*(ω_*k*_) is a multiple of the spectral density, its variance [which depends on *f*(ω_*k*_)] also changes across the frequencies ω_*k*_. To stabilize the variance across frequencies and to standardize comparisons of median curves across two phases (early vs. late phases of the resting-state EEG recording) we will use the log transformed periodograms. It is convenient then, that the variance of the log periodograms at each frequency is constant and takes the approximate value of $\frac{\pi^{2}}{6}$. Moreover, while the periodogram is approximately unbiased for the spectrum, the log periodogram is no longer (approximately) unbiased for the log spectrum due to Jensen's inequality. This is easily fixed by adding the Euler Mascheroni constant 0.57721 to log transformed periodograms to obtain the log bias-corrected periodograms (Wahba, 1980). Let *g*(ω_*k*_) be the true log spectrum, then *Y*_*r*_(ω_*k*_), the log bias of the corrected periodogram at epoch *r*, is defined as

$$Y_{r}(\omega_{k})=g(\omega_{k})+0.57721,\;k=0,1,\ldots ,T/2.$$

**Figure 5:**
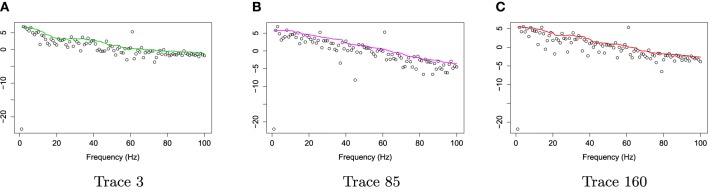
**Log bias-corrected periodograms of epochs 3, 85, and 160 from Channel 197 (Right pre-motor region)**. **(A)** Log bias-corrected periodograms of epoch 3. **(B)** Log bias-corrected periodograms of epoch 85. **(C)** Log bias-corrected periodograms of epoch 160.

Figure 5 gives the log bias-corrected periodograms, *Y*_*r*_(ω_*k*_), corresponding to Figure 4. Throughout this paper, we will apply the gamma crossvalidation method to obtain the optimal smoother of log bias-corrected periodograms.

### 2.4. Functional boxplots

The FBP is constructed in a similar manner to the classical (pointwise) boxplot. Each observation will be sorted based on decreasing values of some depth measure, and band depth is one notion. A curve is said to be “deeply situated” within a sample of curves if it is covered by many bands from pairs of curves. This idea is an extension of a pointwise boxplot where the median is also located “deep” in a sample because it is situated in the middle of the boxplot and hence covered by many pairs of points. Here, our observation units are curves (or real-valued functions) which are the log bias-corrected periodograms *Y*_*r*_(ω_*k*_), *k* = 0, …, *T*∕2 over many epochs *r*. The notion of a band depth was introduced in López-Pintado and Romo (2009) through a graph-based approach to order all sample curves which we briefly describe. Suppose that a curve *Y*(ω_*k*_) is the subset of the plane *G*(*Y*(ω_*k*_)) = {(ω_*k*_, *Y*(ω_*k*_)) : ω_*k*_ ∈ 𝒜 = [0, *T*∕2]}. The band in ℝ^2^ can be delimited by a number *J* of curves, and this number is fixed as *J* = 2 in our study. Now, let *Y*_α_, *Y*_β_ be two continuous functions, *L*_*k*_ = min(*Y*_α_(ω_*k*_), *Y*_β_(ω_*k*_)), and *U*_*k*_ = max(*Y*_α_(ω_*k*_), *Y*_β_(ω_*k*_)). Then the band delimited by *Y*_α_, *Y*_β_ is

$$B(Y_{\alpha },Y_{\beta })=\left((\omega_{k},Y^{\prime }(\omega_{k})):\omega_{k}\in 𝒜,L_{k}\leq Y^{\prime }(\omega_{k})\leq U_{k}\right).$$

Let *Y*_1_, …, *Y*_*n*_ be *n* independent sample curves, then the band depth for a given curve *Y*_*i*_, *i* = 1, …, *n* is defined as

$$BD(Y_{i})={\left(\begin{array}{c} n\\ 2 \end{array}\right)}^{-1}\sum_{\alpha =1,\ldots ,n;\;\beta =1,\ldots ,n}ℐ\left\{G(Y_{i})\subseteq B(Y_{\alpha },Y_{\beta })\right\}$$

where ℐ(·) is the indicator function. When *J* = 2, there are (n2) possible bands delimited by two curves. The limit of the band depth *BD* is that it does not measure the proportion of curve inside the band. Thus, López-Pintado and Romo (2009) also proposed a modified band depth method (MBD), which measures the proportion of a curve *Y*_*i*_ that is actually in a band:

$$MBD(Y_{i})={\left(\begin{array}{c} n\\ 2 \end{array}\right)}^{-1}\sum_{\alpha =1,\ldots ,n;\;\beta =1,\ldots ,n}\lambda \left\{A(Y_{i};Y_{\alpha },Y_{\beta })\right\}$$

where *A*(*Y*_*i*_; *Y*_α_, *Y*_β_) ≡ {ω_*k*_ ∈ 𝒜 : *L*_*k*_ ≤ *Y*_*i*_ ≤ *U*_*k*_}, λ(*Y*_*i*_) = λ(*A*(*Y*_*i*_; *Y*_α_, *Y*_β_))∕λ(𝒜), and λ is a Lebesgue measure on 𝒜. We notice that the MBD computation will be time-consuming when *n* is large, so we use an exact fast method from Sun et al. (2012) to compute the MBD for the EEG data.

Based on the ranks of the depths of the curves, the FBPs can provide the descriptive statistics, such as the 50% central region, the median curve, and the maximum and minimum non-outlying curves. Moreover, the potential outliers can be detected by the 1.5 times inter-quartile range (IQR) empirical rule, which is commonly used for classical boxplots. The boundary region is defined as 1.5 times the height of the 50% central region. Any curves outside this region are considered potential outliers. In contrast with a constant factor 1.5 in classical boxplot, a factor 1.5 in FBP can be modified due to potential spatio-temporal outliers. This is because the curves from different locations will be spatially correlated, and there can be dependence in time/frequency for each curve (Sun and Genton, 2012a).

### 2.5. Surface boxplots

Similar to FBPs, one can compute the data depth of all the observations, then order them according to decreasing depth values. Suppose that the observed sample surfaces, *z*_1_(*s*), …, *z*_*n*_(*s*), *s* ∈ 𝒮, where 𝒮 is a region in ℝ^2^. The information unit for such a dataset is the entire surface. To order sample surfaces, we need to generalize univariate order statistics to surfaces. To this end, we generalize the MBD with *J* = 2 to ℝ^3^ through a volume. Genton et al. (2014) define the sample modified volume depth (MVD) to be

$$MVD_{n}(z)={\left(\begin{array}{c} n\\ 2 \end{array}\right)}^{-1}\sum_{1\leq i_{1}\leq i_{2}\leq n}\lambda_{r}A(z;z_{i_{1}},z_{i_{2}}),$$

where $A\left(z;z_{i_{1}},z_{i_{2}}\right)\equiv \text{s}\in 𝒮:\underset{r=i_{1},i_{2}}{\min}$ $z_{r}\left(\text{s}\right)\leq z\left(\text{s}\right)\leq \underset{r=i_{1},i_{2}}{\max}z_{r}\left(s\right)$ and $\lambda_{r}\left(z\right)=\frac{\lambda \left(A\left(z;z_{i_{1}},z_{i_{2}}\right)\right)}{\lambda \left(𝒮\right)}$, if λ is the Lebesgue measure on ℝ^3^. A sample median surface is a surface from the sample with the largest sample MVD value, designed by argmax_*z*∈*z*_1_, …, *z*_*n*__ *MVD*_*n*_(*z*). If there are ties, the median will be the average of the surfaces maximizing the sample MVD.

The first step for constructing surface boxplots is the surface ordering. Sample surfaces are ordered from the center outwards based on their MVD values, inducing the order *z*_[1]_, *z*_[2]_, …, *z*_[*n*]_. The sample α central region is naturally defined as the volume delimited by the α proportion (0 < α < 1) of the deepest surfaces. In particular, the sample 50% central region is

$$C_{0.5}=\left\{\left(s,z(s)):\underset{{}_{r=1,\ldots ,[n/2]}}{\min}z_{\left[r\right]}(s)\leq z(s)\leq \underset{{}_{r=1,\ldots ,[n/2]}}{\max}z_{\left[r\right]}(s\right)\right\},$$

where [*n*∕2] is the smallest integer not less than *n*∕2. The border of the 50% central region is defined as the inner envelope representing the box in a surface boxplot. This is the surface analog of the first and third quartiles of the classical boxplot. The median surface in the box is the one with the largest depth value. Because the ordering is from the center outwards, the volume of the central region increases as α increases. Hence, the maximum envelope, or the outer envelope, is defined as the border of the maximum non-outlying central region. To determine this region, we propose to identify outlying surfaces by an empirical rule similar to the 1.5 times the 50% central region rule in a FBP. The fences (or the upper and lower surface boundaries for flagging potential outliers) are obtained by inflating the inner envelope (as defined above) by 1.5 times the height of the 50% central region. Any surface crossing the fences are flagged as potential outliers. The factor 1.5 can be also adjusted as in the adjusted FBPs to take into account spatial autocorrelation and possible correlations between surfaces.

### 2.6. Testing for differences in median between families of curves or surfaces

To compare the median curves from two populations of curves, López-Pintado and Romo (2009) proposed the rank sum test. Let ${\tilde{\mu }}_{Y}$ and ${\tilde{\mu }}_{Y^{\prime }}$ be the median curves of two populations *Y* and *Y*′, respectively. Define the null hypothesis to be

$$H_{0}:{\tilde{\mu }}_{Y}={\tilde{\mu }}_{Y^{\prime }}\text{ for all }\mu .$$

Suppose that we observe two sets of curves, namely {*y*_1_, …, *y*_*n*_} and $\left\{y_{1}^{\prime },\ldots ,y_{m}^{\prime }\right\}$. Then define the reference sample to be {*r*_1_, …, *r*_*k*_} which is from one of the two observed sets with *k* ≥ max(*n, m*). The position of a particular *y*_*i*_ for *i* = 1, …, *n*, or $y_{j}^{\prime }$ for *j* = 1, …, *m* with respect to the reference sample *r*, is defined as

$$\begin{array}{l} R(y_{i})=\frac{1}{n}\sum_{l=1}^{n}ℐ\{MBD(z_{l})\leq MBD(y_{i})\},\\ R(y_{j}^{\prime })=\frac{1}{m}\sum_{l=1}^{m}ℐ\{MBD(z_{l})\leq MBD(y_{j}^{\prime })\}, \end{array}$$

where *MBD* is the MBD defined in previous section, and ℐ is the indicator. Then, we can order the values *R*(*y*_*i*_) and $R\left(y_{i}^{\prime }\right)$ from the smallest to the largest, and their ranks are between 1 and *n*+*m*. The test statistics $T=\overset{m}{\underset{l=1}{\sum }}\mathrm{rank}R\left(y_{j}^{\prime }\right)$, then under the null hypothesis *H*_0_, the distribution of *T* is the distribution of the sum of *m* numbers that are randomly chosen from 1, 2, …, *n* + *m* (Sun and Genton, 2012b).

### 2.7. Remarks on the applications of functional and surface boxplots

In this paper, we use functional and surface boxplots to explore the structure of EEGs. However, these methods are general and can be applied to other types of data such as growth data and climate time series (Sun and Genton, 2012b).

## 3. Simulation study

The purpose of the simulation study is to examine the performance of the exploratory spectral methods under various experimental settings. In Section 3.1, we demonstrate the performance of the FBP on the smoothed log periodograms of a mixture of two first order AR time series, denoted AR(1). In Section 3.2, we illustrate the rank sum test to compare the functional median from two families of curves.

### 3.1. Functional boxplot simulation study

For the *r*^*th*^ epoch, let *U*_1*r*_(*t*) be an AR(1) process with its spectra dominated by high frequencies and *U*_2*r*_(*t*) be another AR(1) with its spectra mostly containing low frequencies. The AR(1) parameters are allowed to vary across epochs. Here, we set *t* ∈ *T* = {1, …, 1000}. We define *X*_*r*_(*t*) to be the mixture of *U*_1*r*_(*t*) and *U*_2*r*_(*t*), such that

$$X_{r}(t)=a_{1r}U_{1r}(t)+a_{2r}U_{2r}(t)$$

where *r* = 1, …, 220, *a*_1*r*_ and *a*_2*r*_ are weighted coefficients of *U*_1*r*_(*t*) and *U*_2*r*_(*t*), respectively. Then, the model for high and low frequency AR(1) processes are defined as

$$U_{\ell r}(t)=\phi_{\ell r}U_{\ell r}(t-1)+W_{rt}$$

where ℓ = 1, 2 and *W*(*t*) is white noise. In this setting, the high and low frequency AR(1) are distinguished by the value of ϕ_ℓ*r*_. For example, for high frequency *U*_1*r*_(*t*), we set ϕ_1*r*_ = 0.9 + ξ_*r*_, where ξ_*r*_ are independent and identically distributed from 𝒩(0, 0.001). Similarly, for low frequency *U*_2*r*_(*t*), we set ϕ_2*r*_ = −0.5 + η_*r*_, and η_*r*_ are also independent and identically distributed from 𝒩(0, 0.001). Here, we need the variance of ξ_*r*_ and η_*r*_ to be small so that it guarantees causality, i.e., ξ_*r*_ ∈ (−1, 1) and η_*r*_ ∈ (−1, 1). Next, we split the 220 subjects into two groups, such that the first group will include both high and low frequency series, *U*_1*r*_(*t*) and *U*_2*r*_(*t*), while the second group will only have the high frequency series *U*_1*r*_(*t*). To split *X*_*r*_(*t*) into two groups, we set the weight coefficients *a*_1*r*_ and *a*_2*r*_ as following

$$\begin{array}{l} a_{1r}\sim 𝒩(10,1)\text{ for }r=1,\ldots ,220\\ a_{2r}\sim 𝒩(5,1),\text{ for }r=1,\ldots ,120,\text{ and}\\ a_{2r}\sim 𝒩(0,0.001)\text{ for }r=121,\ldots ,220. \end{array}$$

**Figure 6:**
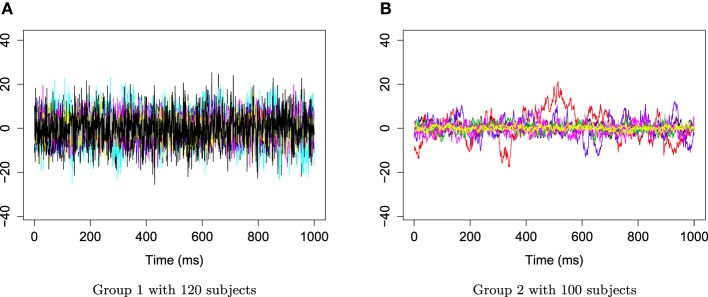
**Time series AR(1) for group 1 and group 2**. **(A)** Time series AR(1) for group 1 with 120 subjects. **(B)** Time series AR(1) for group 2 with 100 subjects.

**Figure 7:**
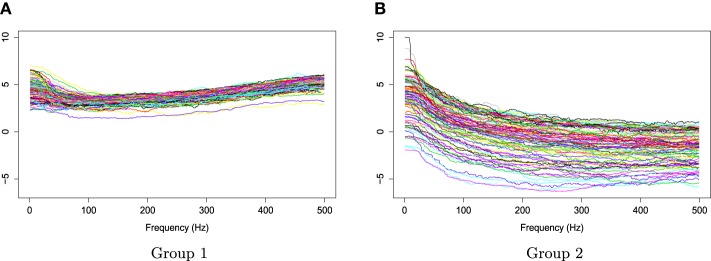
**Smoothed log bias-corrected periodograms for Group 1 and Group 2**.

**Figure 8:**
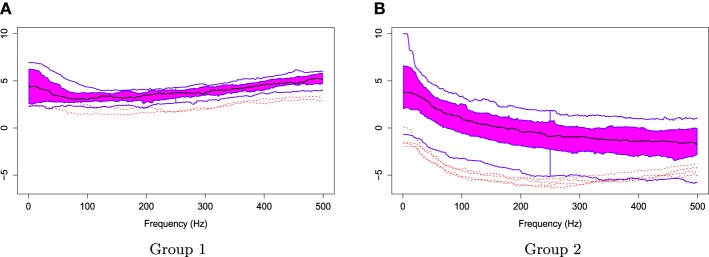
**Functional boxplots of Group 1 and Group 2 with a black curve representing the median curve, the pink area denoting the 50% central region, the two inside blue curves indicating the envelopes of 50% central region, the two outside blue curves representing for two non-outlying extreme curves, and the red dashed curves illustrating the outlier candidates detected by 1.5 times the 50% central region rule**. **(A)** Functional boxplots of Group 1. **(B)** Functional boxplots of Group 2.

The two groups of *X*_*r*_(*t*) are shown in Figure 6. Using the gamma generalized crossvalidation method, Figure 7 displays the log bias-corrected periodograms for each group, and Figure 8 shows the corresponding FBPs. Note that group 1 is dominated by both high (right) and low (left) frequencies while group 2 includes only low frequencies. Thus, the functional median of group 1 should have two peaks, one each in high and low frequency ranges, while the functional median of group 2 has only one peak in the low frequency range. In Figure 8, the black curve is the median curve in the center of the FBP. The two median curves from each group have clearly summarized the typical power distribution for each group. The blue curves in the center form the envelope of the 50% central region. The blue curves outside of the 50% central region are the non-outlying minimum and maximum curves. It is worth remarking that the envelope of group 1 is smaller than the envelope of group 2, and therefore, we demonstrate that group 2 has more dispersion than group 1. Moreover, the envelope of group 1 is in the middle of the non-outlying minimum and maximum curves, while the envelope of group 2 tends to move upwards. This indicates that group 2 shows more skewness than group 1. The red dashed curve in Figure 8 denotes the outliers. We see that the curves from group 1 that are dominated by high frequencies only are detected as outliers while the curves from group 2 that include both high and low frequencies are detected as outliers.

**Figure 9:**
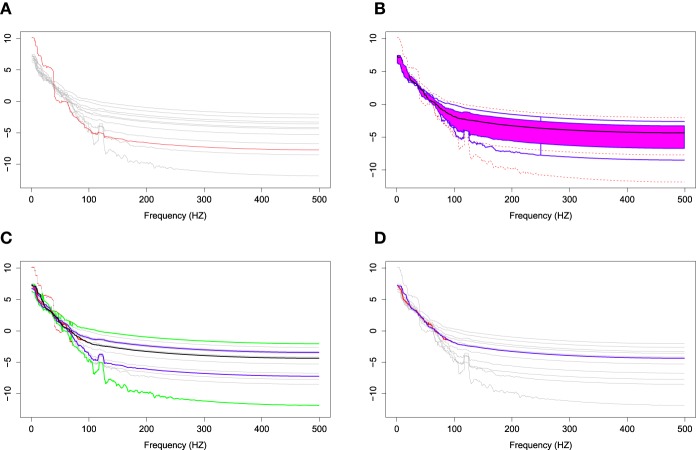
**(A)** Simulation data with gray curves representing sample curves, a red curve denoting the simulated outlying curve **(B)** functional boxplots, **(C)** pointwise boxplots with black curve representing a mean curve, blue curves for the envelope of the 50% central region, the green curves for the non-outlying minimum and maximum curves, and the red points for outliers, and **(D)** two median curves obtaining by functional boxplots method (blue) and pointwise boxplot method (red) are shown in the same plot.

In order to illustrate the usefulness of the FBP compared to the pointwise boxplot, we introduce a simulation study which randomly chooses 10 bias-corrected log periodograms among 160 total periodograms. We simulate an outlying curve by adding additional noise across the 0–100 Hz frequency range, and close to the center for the remaining frequencies. Figure 9A shows the simulation data including the 10 random bias-corrected log periodograms (gray curves) and a simulated outlying curve (red curve). In Figure 9B, the FBP successfully detects the simulated outlying curve and other outliers. However, Figure 9C shows that the pointwise boxplot fails to detect the simulated outlying curve, and provides some disconnected outlying curves across frequencies. We also notice that the non-outlying maximum and minimum curves of pointwise boxplot are actually the outlying curves detected by FBP. Figure 9D compares the two median curves from these two methods, and by visual inspection, there is a slight difference between the two median curves at low frequencies. Thus, FBP can be a non-parametric method to obtain the median curve and the variability around it for EEG data compared to pointwise boxplot.

### 3.2. Rank sum test simulation study

To investigate the performance of this nonparametric test, we simulated two sets of curves, which are defined as below:

$$Y_{\ell ,r}(\omega_{k})=f_{\ell }(\omega_{k})+a_{r}g(\omega_{k})+h_{r}(\omega_{k}),$$

where *r* = 50, ℓ = 1, 2, *g*(ω_*k*_) = 1 for all ω_*k*_, and ω_*k*_ is defined as ω_*k*_ = *k*∕100, where *k* = 1, …, 100. In the model, *f*_1_(ω_*k*_) and *f*_2_(ω_*k*_) are the mean functions; $a_{r}\overset{iid}{\sim }N\left(0,5\right)$ and $h_{r}\left(\omega_{k}\right)\overset{iid}{\sim }N\left(0,2\right)$ represent the variation between and within the curves, respectively. Let the function *f*_1_(ω_*k*_) be defined as

$$f_{1}(\omega_{k})=5\cdot \sqrt{1000\cdot \omega_{k}},$$

and consider three different cases:

1. The two means are identical, let *f*_2_(ω_*k*_) = *f*_1_(ω_*k*_) for all ω_*k*_.
2. There is a slight deviation between the two means; define $f_{2}\left(\omega_{k}\right)=5\cdot \sqrt{900\cdot \omega_{k}}.$
3. There is an appreciable deviation between the two means; let *f*_2_(ω_*k*_) = *f*_1_(ω_*k*_)+2*k*∕3.

**Figure 10:**
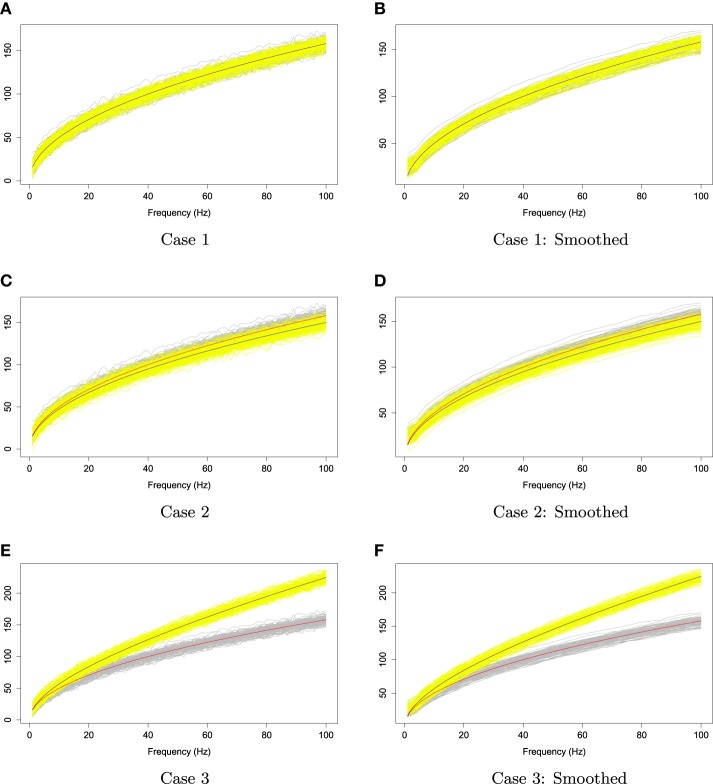
**The two families of simulated curves, ***Y***_1, ***r***_ and ***Y***_2, ***r***_**. The gray shaded area represents the first family, and the yellow shaded area is for the second family. The red and blue lines are the first and second mean functions, *f*_1_ and *f*_2_, respectively.

We applied the kernel average smoother with window size 7 to smooth each curve from these two families. Figure 10 illustrates the simulated curves (left panel) and the smoothed curves (right panel). In order to investigate the rank sum test performance in each case, we simulated two families of curves and obtain *p*-values of rank sum test; this procedure was repeated 1000 times. Let the type *I* error α be 5%, we report the percentage of time that the rank sum test rejects *H*_*o*_ : *f*_1_(ω_*k*_) = *f*_2_(ω_*k*_) for all ω_*k*_ in Table 1.

**Table 1:** **Rank sum test study result**.

	**First case**	**Second case**	**Third case**
Percentage of time rejecting *H*_*o*_	44	605	1000

Overall, the rank sum test method performed well in each case. When the two families are identical, this method rejected the null hypothesis of equality only 44 times (4.4%) out of 1000 times, which is close to the nominal α. When the two families are nearly identical, this method rejects 605 times (the power is 60.5%), and when the two families are completely different, the power is 100%. Thus, this method demonstrates power and sensitivity to differences.

## 4. Analysis of resting-state EEGs data

### 4.1. Data description

In this paper, we analyze EEG data from one participant in a resting-state EEG study approved by the Institutional Review Board of the University of California, Irvine. The over-arching aim of this study was to identify a pattern of EEG-derived coherence acquired during rest-state that could predict subsequent response to training on a novel motor skill. During EEG acquisition, subjects sat quietly with both feet flat on the floor, and were instructed to fixate their gaze to the center of a fixation cross. Each recording was 3 min in duration. While the original EEG recording included 256 channels, only 194 were used in subsequent analyses, as extra-brain artifacts, including cheek and neck muscle artifacts, and heart rhythms, are more likely to contaminate EEG signals recorded from electrodes overlying cheek and neck regions. Following data acquisition, pre-processing steps included: 100 Hz low pass filter; EEG segmentation into 1-s consecutive, non-overlapping epochs; mean detrend; and EEG signal re-reference to mean signal across all 194 channels. In addition, a combination of visual inspection and Infomax Independent Component Analysis decomposition were used to remove extra-brain artifacts, including eye blinks, eye movements, muscle artifact, and heart rhythm artifacts. The final dataset consisted of 160 epochs, with each epoch lasting 1 s, and *T* = 1000 time points for each epoch.

The goals of the present analysis are as follows: In Section 4.2, we closely examined a representative channel in the pre-motor region (specifically channel 197 in this dataset). Since EEGs are not well-localized in space (as opposed to local field potentials), conclusions are constrained to the sensor space. However, electrical activity captured in channel 197 reflects activity roughly around the pre-motor area. Specifically, we estimated the (log) spectrum for each epoch to identify any frequency bin or frequency band that accounts for the majority of the power spectrum. Moreover, using the method of estimating the functional medians, we obtained an estimate of the median curve from the log periodogram curves obtained from several epochs. The median curve is interpreted as a “typical” (log) spectral profile across several epochs. Using this method, we also identified outlier curves which could also be interpreted as epochs with “unusual” EEG activity. In Section 4.3, we investigated the possibility of non-stationarity across the 3 min resting-state EEG recording. Our specific goal was to compare the log spectrum during the early phase (first 60 epochs) of the recording with the log spectrum during the late phase (last 60 epochs) of the recording, and identify frequency bands that exhibit any differences between the early vs. late phases. In Section 4.4, we studied the spatial variation of power, at each of the five frequency bands: delta, theta, alpha, beta, and gamma, across all 194 channels, with the goal of identifying regions that exhibit relatively greater proportion of spectral power in each of the five frequency bands of interest. Finally, we compared the spatial variation for each of the five bands during the early vs. late phases of the resting-state EEG recording.

### 4.2. Functional medians of the pre-motor log spectral curves

**Figure 11:**
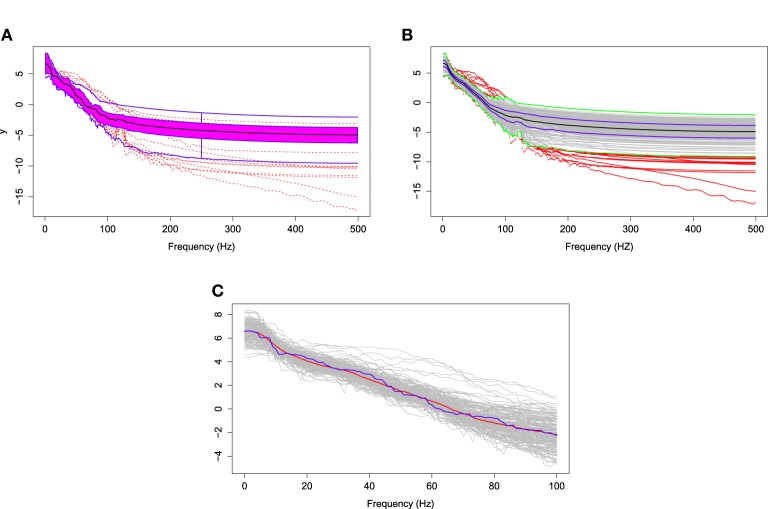
**(A)** The functional boxplots, **(B)** pointwise boxplots of log bias-corrected periodograms, and **(C)** two median curves obtaining by functional boxplot method (blue) and pointwise boxplot method (red) are shown in the same plot.

The log of the bias-corrected periodograms at the representative channel (channel 197) that approximately overlies cortex of the pre-motor region recorded for several traces and the FBPs are displayed in Figure 11A. The functional median curve is represented by the black curve, which is located inside the 50% central region, shaded area. The two blue curves outside of the shaded area are the non-outlying maximum and minimum curves. Similar to a FBP, we show in Figure 11B the pointwise boxplot (per frequency point), where the black curve is the median obtained by connecting the medians at each frequency point; the blue curves form the central region (50-th percentile region); the green curves are two non-outlying extreme curves. We compared these two median curves in Figure 11C and noted a slight discrepancy between these median curves derived using a FBP and the pointwise boxplot, with an emphasis on the low frequency range. The main difference between the functional median and the point-wise median curve is in the interpretation. The former is one of the curves from a recorded epoch, whereas the latter may not be an actual curve. Hence the latter cannot really be interpreted as a “typical” curve from a family of curves formed from several epochs. Moreover, the FBPs approach allows us to identify specific epochs that produce “unusual” or outlying log bias-corrected periodogram curves. Note that in the plots, the gray curves are the log bias-corrected periodograms of 160 epochs and the red curves are outliers. Figure 11B also shows that these outlying curves are discontinuous around the frequency bin centered at 100 Hz.

### 4.3. Testing for stationarity of EEG epochs across the entire resting-state

In the previous section, the FBP provided descriptive statistics for the log bias-corrected periodograms of 160 epochs from the pre-motor region. Note that there were originally 180 epochs but 20 had to be removed from further analysis due to extra-brain artifact contamination. Our interest now is to test whether resting-state brain activity evolved across the 3 min EEG recording. While there are many ways to characterize such an “evolution” of the underlying brain processes, here we will specifically look into changes on the log spectral curves for early vs. late phases of the resting-state EEG recording. In this case, a change in the log spectral power in early vs. late phases would indicate non-stationarity of the EEG signal across the resting-state recording.

**Figure 12:**
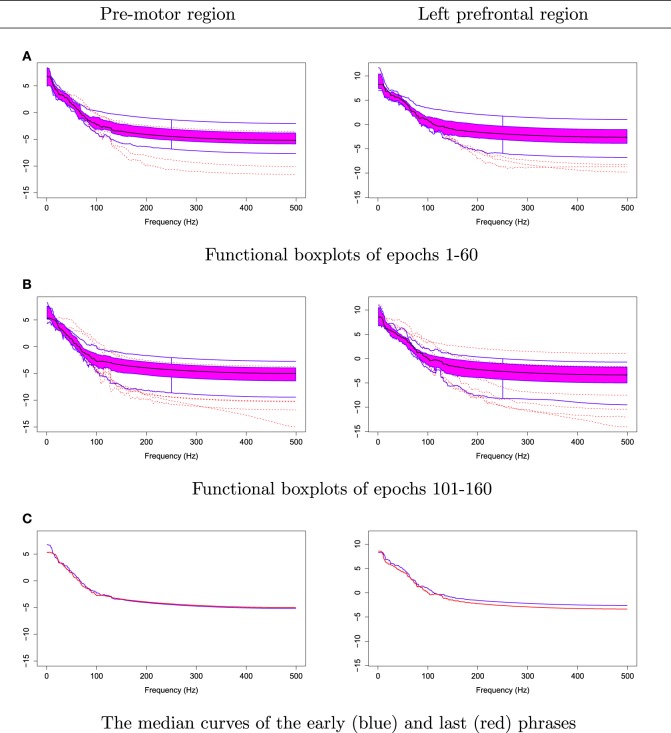
**Comparing median curves of the early and last phrases from pre-motor region and left pre-frontal region**.

The null hypothesis of stationarity here is that the true median curves of the early and last phrases are identical. We test this hypothesis using the rank sum test with the significance level set to 0.05. We defined the early phase to include the first 60 epochs (60 s) of the 3 min recording and the late phase to include the last 60 epochs. In Figure 12, we display the FBPs and the other descriptive statistics for each phase. A visual inspection suggests that the median curves are only slightly different from each other for electrodes that approximately overlie the pre-motor region. More significant differences are noted for electrodes that approximately overlie the prefrontal region (see Figure 12C). Moreover, the rank sum test failed to reject the null hypothesis, as the *p*-value is 0.56. Therefore, the two median curves are not significantly different and the hypothesis of stationarity in the pre-motor regions is not rejected. This is not entirely unexpected since the whole 3-min recording was purely resting-state. There was no experimental stimulus and the time frame was short.

**Figure 13:**
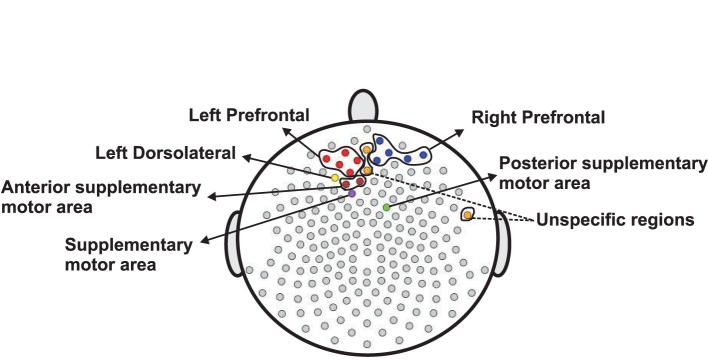
**Color circles represent channels, which have significant difference between the median curve of first 60 epochs and the median curve of last 60 epochs at α = 0.05**. Gray circles represent channels which do not have significant difference between the median curve of first 60 epochs and the median curve of last 60 epochs at α = 0.05.

Next, we use the same testing procedure at this particular channel in the pre-motor region (channel 197) to test the same null hypothesis of non-evolution of the brain process at each of the other channels across the 3 min EEG recording. Among the 194 total channels, 18 channels were identified that demonstrated a significant difference in median curves during the early vs. late phase at a significance level of 0.05. These channels are represented by colored circles in Figure 13. Of these 18, channel 29 (approximately overlying the supplementary motor area) has the lowest *p*-value at 10^−4^. Since we repeat the same test for 194 channels, we used the Bonferroni correction so that the significance level for each test was set to 0.05∕194 = 2 × 10^−4^. Indeed, only channel 29 (anterior supplementary motor area) survived the stringent threshold after the Bonferroni correction.

The tests for temporal stationarity at each channel (local spatial tests) revealed several channels having a significant difference between the median curves of the early vs. last phases of the EEG recording. As a next step, we studied stationarity in each of 19 predefined regions of the cortex. In this analysis, the representative EEG signal for each region was obtained by averaging the EEG signal-epochs over all channels within each region.

**Figure 14:**
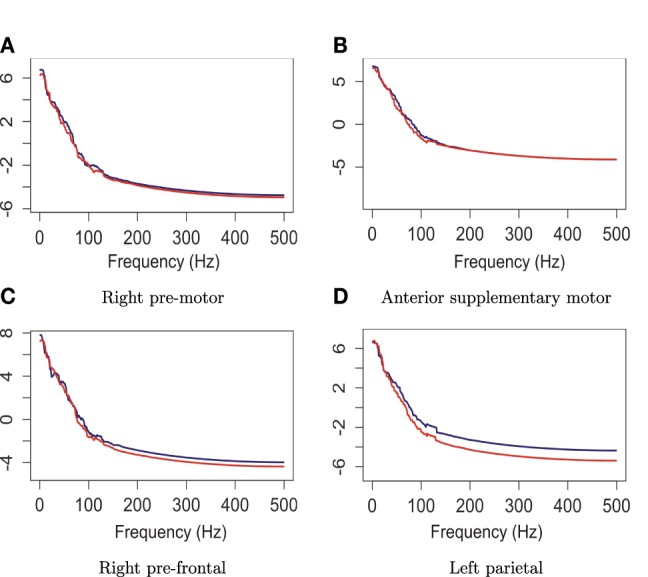
**Median curves of the early phase (first 60 epochs, in blue) and the late phase (last 60 epochs, in red) in the right pre-motor, anterior supplementary motor, right pre-frontal and left parietal regions**.

**Figure 15:**
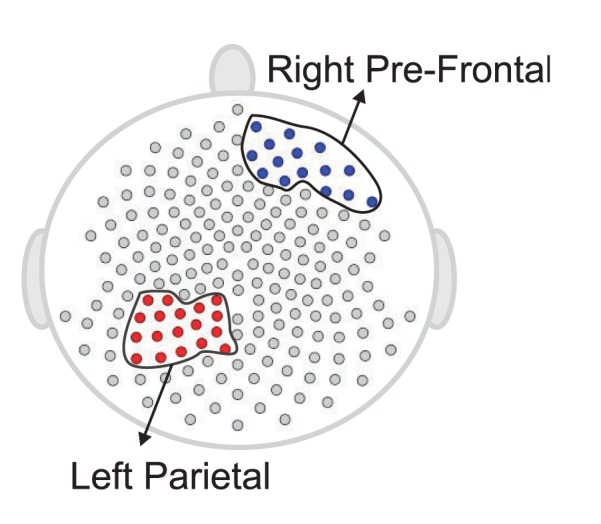
**Testing for difference between the early and late phases of the resting-state for each region**. The right pre-frontal regions (blue circles) and the left parietal regions (red circles) exhibit significant non-stationarity at level 0.05.

The plots in Figure 14 suggest that the median curves for the early vs. late phases of the EEG recording are similar for EEG signals recorded from channels that approximately overlie right pre-motor and anterior supplementary motor regions, but different in the right pre-frontal and left parietal regions. Indeed, we conclude from the rank sum test that there is significant difference between the early vs. late phases in cluster of channels that approximately overlie the right pre-frontal (*p* = 0.01) and the parietal regions (*p* = 0.029). We found that the right pre-frontal region is significantly non-stationary (i.e., early and late phases differ) at level 0.05 (see Figure 15). This result overlaps with the channel-specific tests, in which several of the channels identified to be non-stationary in the single channel tests are included in the predefined right pre-frontal region. In contrast, while the cluster of electrodes that overlie the left parietal region was found to be non-stationary in the region-by-region tests, none of the 18 channels that were identified to be non-stationary in the single channel tests are part of the left parietal cluster. Therefore, the additional averaging step across group of channels may improve signal-to-noise in this type of analysis. A similar phenomenon was also noted for predefined clusters of electrodes overlying at the left pre-frontal region.

### 4.4. The variation of spectral power at each frequency band across the entire cortex

**Figure 16:**
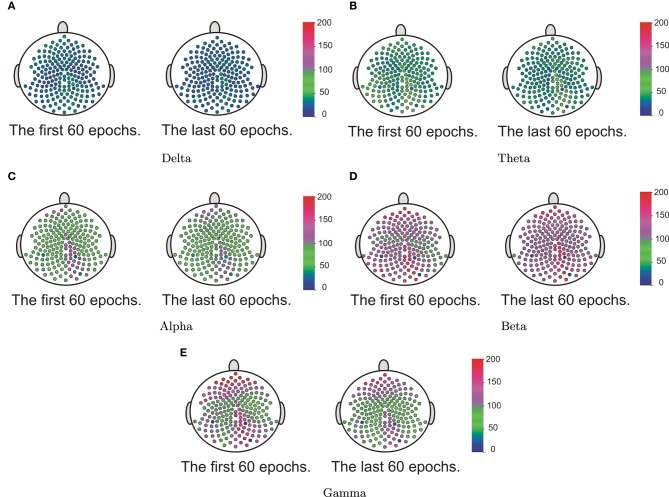
**The median surfaces of five frequency bands**.

Our goal here is to test whether the spectral power at each frequency band differed across the cortical surface. We first computed the estimate of the spectral power for each channel at each epoch. Starting with the delta band, for each epoch we construct a 2 − *D* surface plot of the delta power across the entire cortical surface of 194 channels. These surfaces were then grouped according to the early and late phases of the resting-state. We then applied the surface boxplot method for each frequency band to obtain the median surfaces. In Figure 16, we present the median surface for five frequency bands in the early and late phases. The color blue represents the low spectral power while red is for high power. In Figure 16, it is interesting that even during resting-state there is relatively high spectral power at the beta and gamma bands—which are both associated with higher cognitive processing (Engel and Fries, 2010).

**Figure 17:**
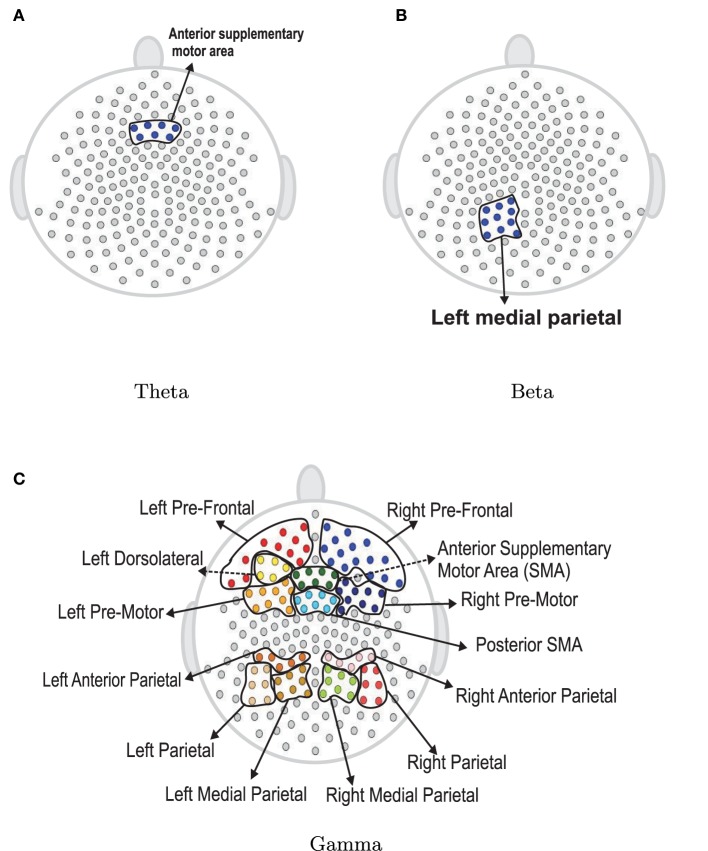
**Rank sum test results for regions**. Color circles in a bounded curve represents a region, which has a significant difference between the first 60 epochs and the last 60 epochs.

The next step is to test for differences between the early and late phases of the EEG recording for each of the five frequency bands of interest. Using the rank sum test, the delta and alpha bands do not have significant difference between the early and late phases. However, theta, beta and gamma bands show significant differences. In Figure 17, the colored regions indicate significant differences between the first and last phases while the gray color regions indicate no significant differences between these two phases. For the theta band, the rank sum test rejected the null hypothesis at only one region which is the cluster of electrodes overlying anterior supplementary motor. For the beta band, the rank sum test identified differences at the left medial parietal region. For the gamma band, there were 13 regions (out of 19) with significant difference between the early and late phases. Since the gamma band is wider than other bands, an estimated spectrum powers' variation across channel in gamma band is expected to be smaller than the estimated spectrum powers' variation in other bands. In Section 4.3, we tested the stationarity for each region. Figure 15 shows two regions, namely, the right pre-fontal and left parietal, which are significantly non-stationary across all frequencies between the early and late phases. Figure 17 shows that the cluster of electrodes overlying the left parietal region exhibits significant non-stationarity in the beta and gamma bands while the cluster of electrodes overlying the right pre-fontal region is significantly non-stationary only in the gamma band.

## 5. Conclusion

This study has extended the use of the classical boxplot to FBP, which is a new visualization tool to analyze functional neuroimaging data, including EEG. The primary findings from the current study demonstrate the FBP is useful for both characterizing the spectral distribution of both simulated and real EEG data and identifying potential outliers in a continuous EEG signal.

In the current implementation of the FBP, ranked sample curves are used to characterize the EEG spectrum by defining a 50% central region, a median curve, and maximum and minimum non-outlying curves. Thus, the shape, size, and length of the FBP can be used to characterize the distribution of the dataset, including the skewedness and degree of variability of the EEG recording. Therefore, potential application of the FBP in this context includes comparing FBPs derived from EEG recordings before and after an experimental intervention (e.g., across a period of motor skill training), comparing mean FBPs derived from EEG recordings in healthy and diseased experimental groups, and comparing mean FBPs derived from EEG recordings during resting-state vs. task.

An additional use of the FBP, as demonstrated by the current results, is to identify potential outliers of the EEG recording. Extra-brain artifacts, including eye blinks, eye movements, heart rhythms measured at pulse points downstream, and muscle movements can cause large deviations in the EEG signal, and represent a significant hurdle in EEG signal processing (Delorme et al., 2007). As a method for identifying outliers in the EEG signal, the FBP could be used to rapidly identify periods of an EEG recording that show high likelihood for contamination by artifacts. In clinical applications, the continuous EEG recording has demonstrated promise as a method for monitoring neural function in patients who have compromised level of consciousness (Fyntanidou et al., 2012) or changes in neural function in patients undergoing neurosurgical interventions (de Vos et al., 2008). The use of FBP to identify outliers in the EEG recording represents a novel method for determining periods of the EEG recording that represent changes in consciousness in patients with a compromised level of consciousness, or for determining changes in neural function across neurosurgical intervention.

The current study also presents an application of the FBP to examine resting-state EEG data acquired from a single individual by comparing EEG signals acquired during early vs. late phases of the 3 min EEG recording. This result has important implications for resting-state studies of neural activity, as many neuroimaging studies that examine resting-state brain function assume resting-state neural activity to be static. However, recent studies that examine dynamic changes in resting-state neural activity suggest momentary change in cognitive processes can cause non-stationarity in resting-state function (Chang and Glover, 2010; Hansen et al., 2015). In contrast, the current results show that the majority of channels demonstrate stationarity across the recording period, and provide support for the assumption that the average EEG signal is static across a 3 min EEG recording. Combined with previous findings, the current results suggest that while momentary changes in cognitive processes result in non-stationary fluctuations of the time series, when averaged across a 60 s subset of the complete 3 min EEG recording, the EEG signal is relatively static. This is supported by the current results that show channels which demonstrate non-stationarity of the EEG signal when comparing early and late phases of the recording include electrodes that overlie the right prefrontal region, which is associated with higher-order cognitive processes (Logue and Gould, 2014). Thus, the assumption of stationarity in resting-state functional neuroimaging studies may be more appropriate for non-cognitive networks, including the motor network. Regardless, further work is needed to determine the minimal time-frame in which EEG signal demonstrate stationarity.

Additional future work is focused on developing a new method for computing confidence bands for the median curve. This method needs to consider the data as a whole. One possible approach is a re-sampling method, in which the notion of band depth is used to construct a 95% confidence band. A potential limitation of the re-sampling method is that there is the potential for multiple curves demonstrating ties with respect to band depth, thus affecting the resultant confidence band. One of the assumptions of the current smoothed periodogram method is that the log bias-corrected periodogram is an unbiased estimator of spectrum. Future work will provide further investigation of this assumption as the current method includes several levels of periodogram manipulation, including smoothing with the gamma generalized crossvalidation, log transformation, and correction by adding Euler Mascheroni constant. In conclusion, the current study presents a novel implementation of the FBP and demonstrates promise as a method for exploratory analysis of complex, high-dimensional neuroimaging datasets, including EEG data.

## Conflict of interest statement

The authors declare that the research was conducted in the absence of any commercial or financial relationships that could be construed as a potential conflict of interest.
